# Simulation of cardiac arrhythmias in human induced pluripotent stem cell-derived cardiomyocytes

**DOI:** 10.1371/journal.pone.0310463

**Published:** 2024-09-27

**Authors:** Thea Bommer, Maria Knierim, Julia Unsöld, Dominic Riedl, Laura Stengel, Michael Paulus, Thomas Körtl, Norman Liaw, Lars S. Maier, Katrin Streckfuss-Bömeke, Samuel Sossalla, Steffen Pabel

**Affiliations:** 1 Department of Internal Medicine II, University Hospital Regensburg, Regensburg, Germany; 2 Department of Cardiothoracic and Vascular Surgery, University Medical Centre Göttingen, Göttingen, Germany; 3 Institute of Pharmacology and Toxicology, University of Würzburg, Würzburg, Germany; 4 Justus-Liebig-University Gießen Medical Clinic I and Campus Kerckhoff Bad Nauheim, Gießen and Bad Nauheim, Germany; 5 Institute of Pharmacology and Toxicology, University Medical Centre Göttingen, Göttingen, Germany; 6 Clinic for Cardiology and Pneumology, Georg-August University Göttingen, DZHK (German Centre for Cardiovascular Research), Partner Site Göttingen, Göttingen, Germany; 7 Center for Systems Biology, Massachusetts General Hospital and Harvard Medical School, Boston, MA, United States of America; University of Minnesota, UNITED STATES OF AMERICA

## Abstract

The effects and mechanisms of cardiac arrhythmias are still incompletely understood and an important subject of cardiovascular research. A major difficulty for investigating arrhythmias is the lack of appropriate human models. Here, we present a protocol for a translational simulation of different types of arrhythmias using human induced pluripotent stem cell-derived cardiomyocytes (hiPSC-CM) and electric cell culture pacing. The protocol comprises the handling of ventricular and atrial hiPSC-CM before and during in vitro arrhythmia simulation and possible arrhythmia simulation protocols mimicking clinical arrhythmias like atrial fibrillation. Isolated or confluent hiPSC-CM can be used for the simulation. In vitro arrhythmia simulation did not impair cell viability of hiPSC-CM and could reproduce arrhythmia associated phenotypes of patients. The use of hiPSC-CM enables patient-specific studies of arrhythmias, genetic interventions, or drug-screening. Thus, the in vitro arrhythmia simulation protocol may offer a versatile tool for translational studies on the mechanisms and treatment options of cardiac arrhythmias.

## Introduction

**Fig 1:**
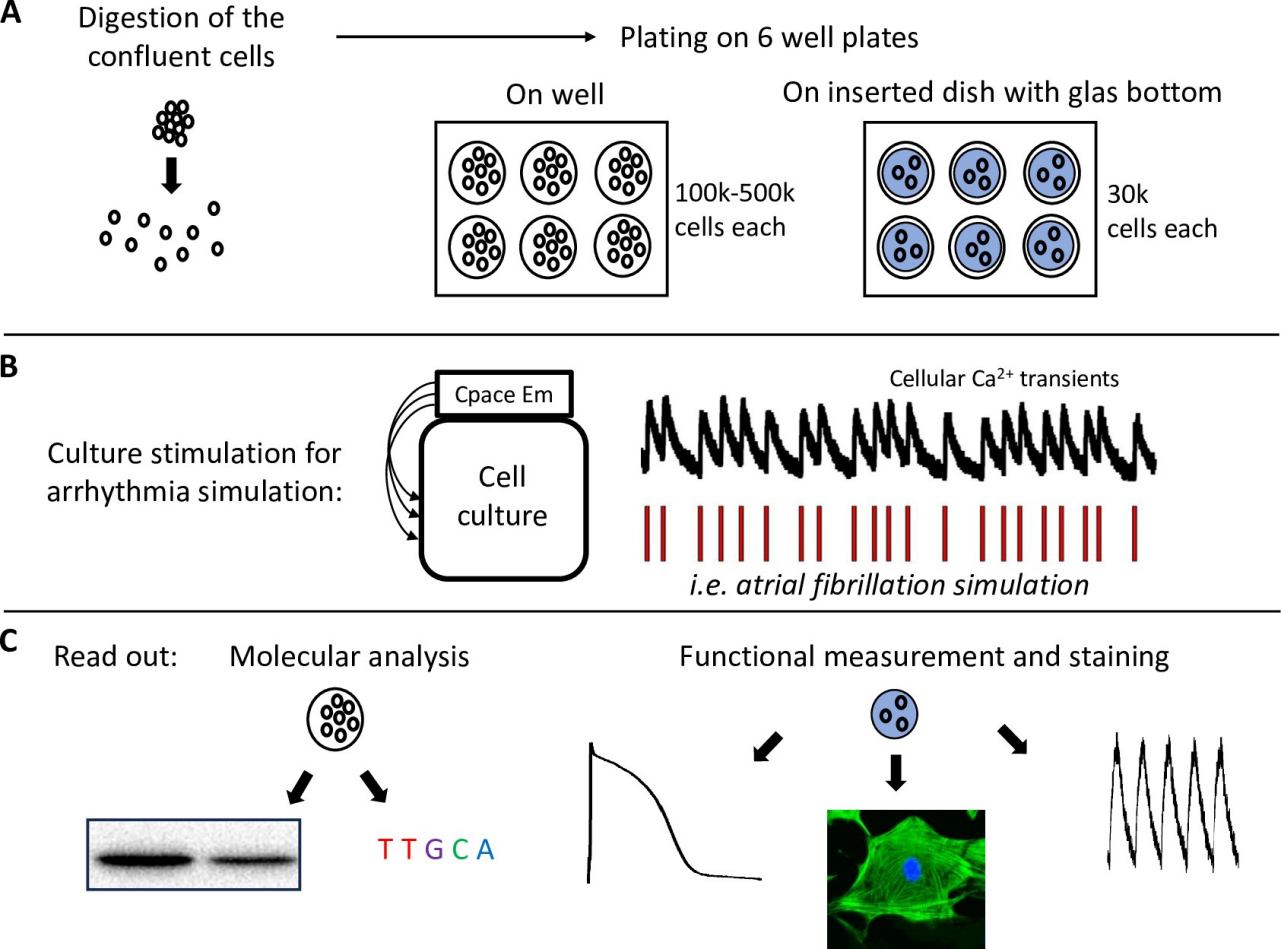
Summary of the protocol for in vitro arrhythmia simulation using hiPSC-CM. **(A)** Confluent grown hiPSC-CM are digested and plated depending on the planned experimental read-out on 6 well plates (100 000–500 000 cells per well i.e. for acquisition of cell pellets) or on glass bottom dishes (30 000 cells per dish inserted in the 6 well plates i.e. for measuring single cells). **(B)** After plating of the cells, the electrodes of the C-Pace EM stimulation system are placed as lid on the 6-well dishes and arrhythmia simulation is conducted. **(C)** After arrhythmia simulation, cells can be directly used for respective experiments.

Arrhythmias significantly determine cardiovascular morbidity and mortality in the western nations [1]. Appropriate management of cardiac arrhythmias is a major medical and epidemiological challenge [1]. The effects and mechanisms of diverse types of atrial and ventricular arrhythmias are a subject of various studies, but remain incompletely elucidated. Even fewer research efforts succeeded in providing novel pharmacological treatment options for clinical application [2]. The number of clinically used antiarrhythmic agents is still small and existing drugs are often associated with severe side effects. Thus, available pharmacological treatment options remain restricted. More defined approaches may be needed that directly target disease-specific pathways thereby enabling individualized therapy. Therefore, there is a clinical need for further mechanistic and pharmacological studies for a better understanding of cardiac arrhythmias [3]. A major difficulty of experimental investigations on the mechanisms and potential treatment options for cardiac arrhythmias are the limitations of preclinical models. Cardiac tissue of patients is of the highest translational importance but limited by restricted availability, methodological difficulties, and patient-specific confounders. Moreover, chronic in vitro drug testing is barely feasible in human myocardium. Due to limited availability, it is even more difficult to reach meaningful sample sizes. Animal models where arrhythmias can be simulated/induced by electrical stimulation or genetic modulations [4–10] may overcome some methodological limitations. However, species differences of (cardiomyocyte) electrophysiology compared to human myocardium and potential off-target effects of genetic/pharmacological modulations limit the translational importance of animal studies [11]. Therefore, it is challenging for basic research studies to translate preclinical findings into clinical application [2]. Human induced pluripotent stem cell-derived cardiomyocytes (hiPSC-CM) are an established, well-accessible, human in vitro cell model suitable for modeling various disease phenotypes and mimicking properties of human cardiomyocytes [12]. Importantly, hiPSC-CM offer the possibility to chronically test effects of drugs in a human based system or to conduct patient specific studies. Here we provide an approach for prospective long-term in vitro simulation of cardiac arrhythmias using ventricular and atrial hiPSC-CM as a human translational model. The protocol describes how to simulate different types of cardiac arrhythmias in cultured hiPSC-CM for consecutive molecular and functional investigations (Fig 1). Key aspects of the arrhythmia simulation include the handling of hiPSC-CM before and during culture pacing, the settings and adjustments of the electrical stimulation as well as the application of different simulation protocols. Ventricular and atrial hiPSC-CM are suitable for various experimental read-outs after in vitro arrhythmia simulation. Based on the broad possibilities of stem cell research, the effects and mechanisms of arrhythmias can potentially be investigated with respect to different cell types, patient-specific disease modeling, genetic modulations and pharmacological treatment. Therefore, this protocol offers a possibility to perform prospective in vitro studies on cardiac arrhythmias in a standardized manner using human-based tissue.

## Materials and method

The step-by-step arrhythmia simulation protocol described in this article is published on protocols.io (https://www.protocols.io/view/simulation-of-cardiac-arrhythmias-in-human-induced-kqdg32k1qv25/v1 or: dx.doi.org/10.17504/protocols.io.kqdg32k1qv25/v1). The protocol on protocols.io is linked to PLOS ONE and is a peer-reviewed method. The protocolis provided as S1 File of this article. All procedures were performed according to the Declaration of Helsinki and were approved by the local ethics committee of the University of Göttingen (ref. no. 10/9/15). Informed written consent was obtained from all participants.

## Contextualization of the protocol

### Electrical excitation of the cells

**Fig 2:**
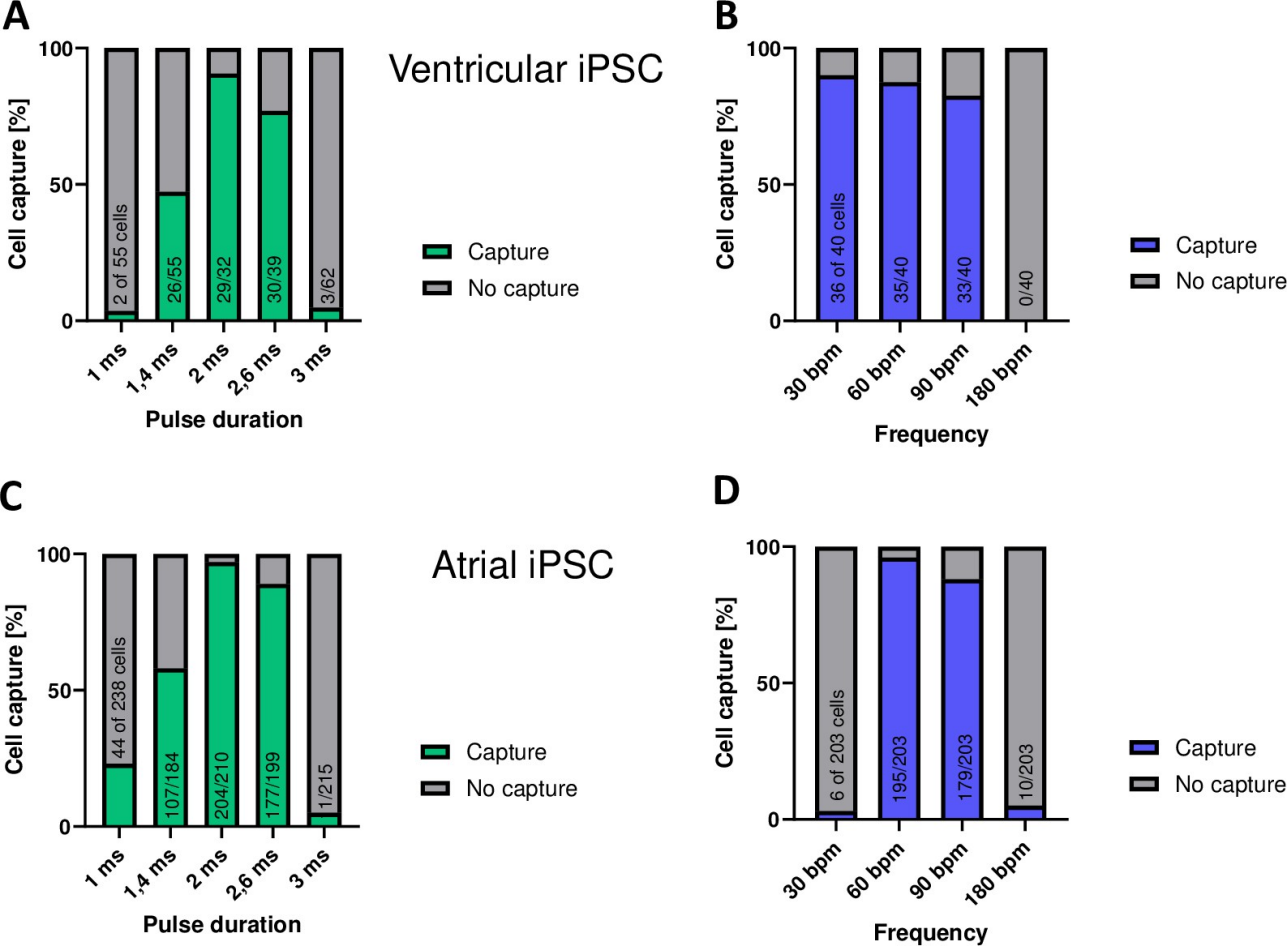
Cell capture of ventricular and atrial hiPSC-CM. **(A)** Part of capturing ventricular hiPSC-CM during stimulation with 1, 1.4, 2, 2.6 and 3 ms stimulation pulse duration and a stimulation pulse amplitude of 20 V at 60 bpm. Cells were stimulated up to 7 days (total counted cells: 55 at 1 ms, 55 at 1.4 ms, 32 at 2 ms, 39 at 2.6 ms, 62 at 3 ms). **(B)** Part of cells capturing the acute stimulation at 30, 60, 90 and 180 bpm after 7 days of culture stimulation at 60 bpm (total counted cells: 40 each). **(C)** Part of capturing atrial hiPSC-CM during stimulation with 1, 1.4, 2, 2.6 and 3 ms stimulation pulse duration and a stimulation pulse amplitude of 20 V at 60 bpm. Cells were stimulated up to 7 days (total counted cells: 238 at 1 ms, 184 at 1.4 ms, 210 at 2 ms, 199 at 2.6 ms, 215 at 3 ms). **(D)** Part of cells capturing the acute stimulation at 30, 60, 90 and 180 bpm after 7 days of culture stimulation at 60 bpm (total counted cells: 203 each).

Setting the electrical stimulus as outlined in the method section is fundamental for appropriate stimulation and contraction of the cardiomyocytes and thus for the induction of the arrhythmia-associated phenotype. When setting the electrical culture stimulation, we found that the stimulus pulse duration is the more critical parameter compared to the pulse amplitude. A long pulse duration more likely resulted in an increased fraction of apoptotic, damaged or detached hiPSC-CM. We tested different combinations of the stimulus duration and amplitude for achieving the balance between sufficient excitation and cell survival during long term pacing. Regarding stimulation duration, we evaluated values around 2 ms (between 1.8 and 2.2 ms) as the best range to fit these requirements. We stimulated ventricular and atrial hiPSC-CM up to 7 days with different stimulation pulse durations (1.0 ms, 1.4 ms, 2 ms, 2.6 ms, 3 ms) and evaluated the fraction of cells with sufficient capture of the stimulus leading to cell contraction (Fig 2A and 2C). When cells were stimulated with a pulse duration of 1.0 and 1.4 ms only 3% (ventricular)/23% (atrial) and 47% (ventricular)/ 58% (atrial) of the cells captured the stimulation. With a stimulus pulse duration of 2 ms 90%/97% of the cells captured rhythmically with visible contraction. Higher pulse durations like 2.6 or especially 3 ms resulted in worse capturing (77%/89% and 5%/5%) in long term stimulation, mainly because of increased detachment or apoptosis. A pulse amplitude of 20–25 V usually comes along for sufficient excitation. A likely explanation for the narrow range of the optimal stimulation pulse duration is that the pulse must be high enough to activate voltage-gated Na^+^ channel to initiate the cardiac action potential and Ca^2+^ induced Ca^2+^ release for sufficient contraction. A too long pulse duration may induce cellular distress. Cellular electrophysiology determines many intracellular processes but requires a lot of energy due to ATP consuming ion pumps [13]. A too long membrane depolarization might cause alterations of subcellular functions, ATP consumption, generation of reactive oxygen species and ultimately induction of proapoptotic processes during chronic pacing.

Pacing was well feasible at faster rates, depending on the differentiation experiments, to induce sufficient capturing of cardiomyocytes and was well tolerated for up to 7–10 days. We stimulated ventricular and atrial cells for 7 days with 60 bpm and counted capturing (Fig 2B and 2D) after this time during stimulation with different frequencies: 30 bpm, 60 bpm, 90 bpm and 180 bpm. The same cells were stimulated and counted at each frequency. Observing the ventricular hiPSC-CM at 30 bpm, 90% of the cells captured, at 60 bpm 88% and at 90 bpm 83% captured each stimulated pulse with visible contraction. At stimulation with 180 bpm no cell captured the high frequency accurately. Counting the capturing of the atrial hiPSC we calculated 3% capturing at 30 bpm. At 60 bpm 96% of the cells captured, at 90 bpm 88% and at 180 bpm 5%. Of note, cell capture and contraction may vary between differentiation experiments and were influenced by cell age, purity and cardiac differentiation efficiency (i.e. as indicated by expression of cardiac markers like cardiac troponin T or myosin light chain 2). Usually, hiPSC-CM could be well identified with visible, although disorganized, striation patterns. Too low stimulation rates (i.e. <30 bpm) might not lead to a 1:1 capture because of higher intrinsic beating rates of hiPSC-CM (especially observed in atrial hiPSC-CM). High stimulation rates (i.e. 180 bpm) might be limited by relaxation time of hiPSC-CM.

### Cell death is not influenced following in vitro arrhythmia simulation

**Fig 3:**
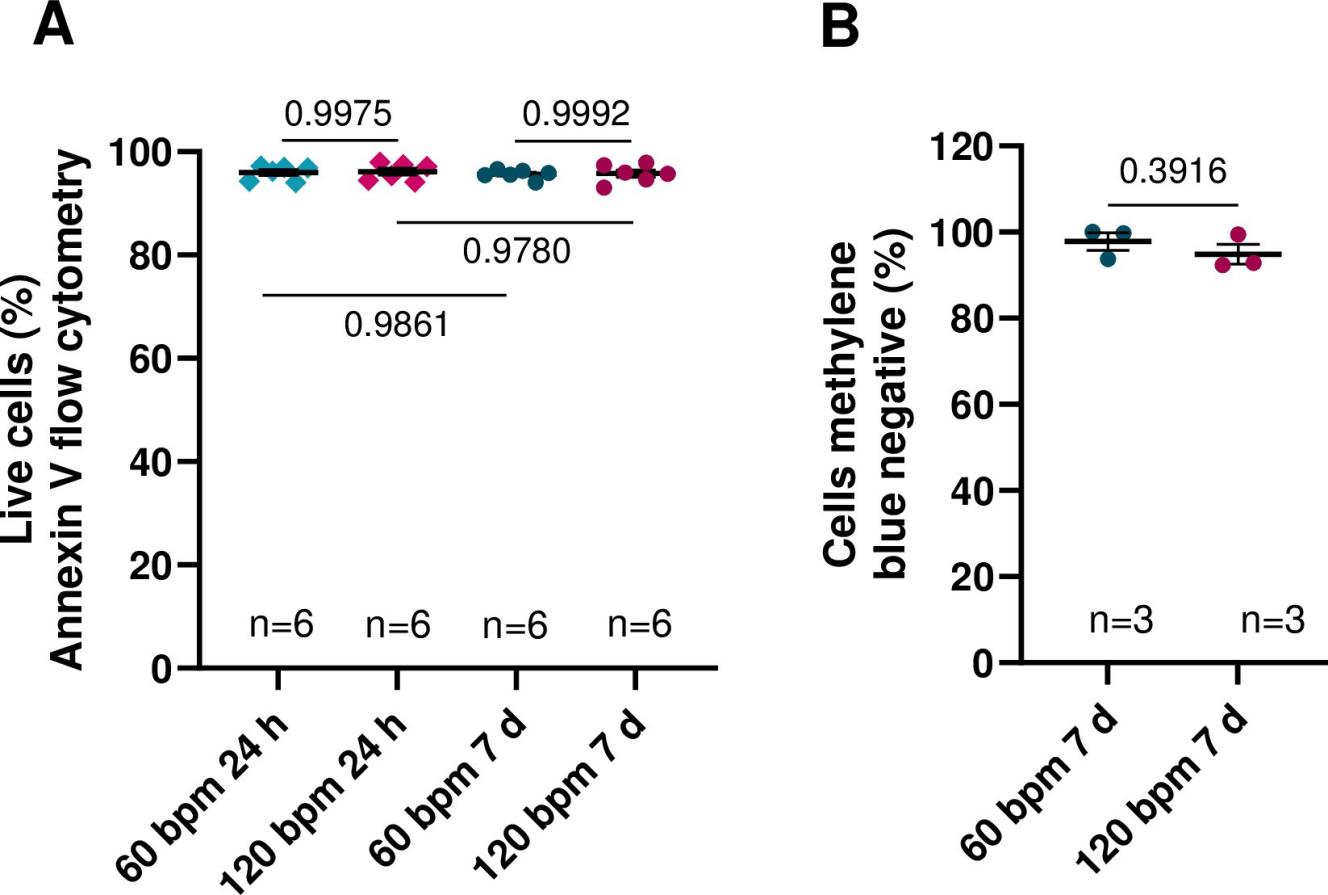
Cell apoptosis after rhythmic (60 bpm) and tachycardic (120 bpm) culture pacing. **(A)** Percentage of living cells measured with flow cytometry (Annexin V) after 24h and 7d culture pacing at 60 bpm or 120 bpm (n = 6 differentiations). **(B)** Percentage of methylene blue negative cells after 7 days culture pacing at 60 bpm and 120 bpm (n = 3 differentiations). Data are shown as scatter plot with mean±SEM, each data point represents one differentiation. P-values were calculated using Student’s t-test.

One important requirement for using cell culture pacing for in vitro arrhythmia simulation is that the culture pacing per se does not damage the cells or induce cell death. We assessed the apoptotic events of hiPSC-CM after 24 h and 7 days chronic culture pacing via Annexin V flow cytometry for apoptosis [14]. The percentage of living hiPSC-CM after 24 h pacing at 60 bpm was 95.9±0.6% and did not differ compared to 24 h pacing at 120 bpm (96.1±0.7%, Fig 3A). The percentage of living cells after 7 days stimulation with 60 bpm was 95.6±0.4% and was not significantly different to the value after 24h stimulation. Also 120 bpm stimulation for 7 days did not cause significantly more cell death (95.8±0.7%) compared to 7 days stimulation at 60 bpm or 24h stimulation at 120 bpm. Moreover, methylene blue stainings were performed to analyze the percentage of intact hiPSC-CM after 7 days culture pacing. Pacing at 60 bpm for 7 days resulted in 97.8±2.9% methylene blue negative cells which did not differ compared to pacing at 120 bpm for 7 days (94.9±2.3%, Fig 3B). Thus, pacing at 60 bpm as well as at 120 bpm was well tolerated with respect to cell survival. In previous work we could demonstrate that chronic in vivo arrhythmia simulation for simulating atrial fibrillation (AF) was well feasible and chronic pacing of hiPSC-CM had no influence on cardiac markers like myosin light chain 2 [15].

### Simulating cardiac arrhythmias

The C-Pace EM system (Ion Optix) allows a variety of individual settings for stimulation protocols, which can mimic clinical arrhythmias (Fig 1B). Importantly, arrhythmia simulation can be used in various patient-specific hiPSC models including atrial or ventricular differentiated cells, genetic modifications as well as pharmacological modulation (i.e. treatment with potential antiarrhythmic agents). In this section, some possible tested arrhythmia protocols are presented.

### Atrial fibrillation

**Fig 4:**
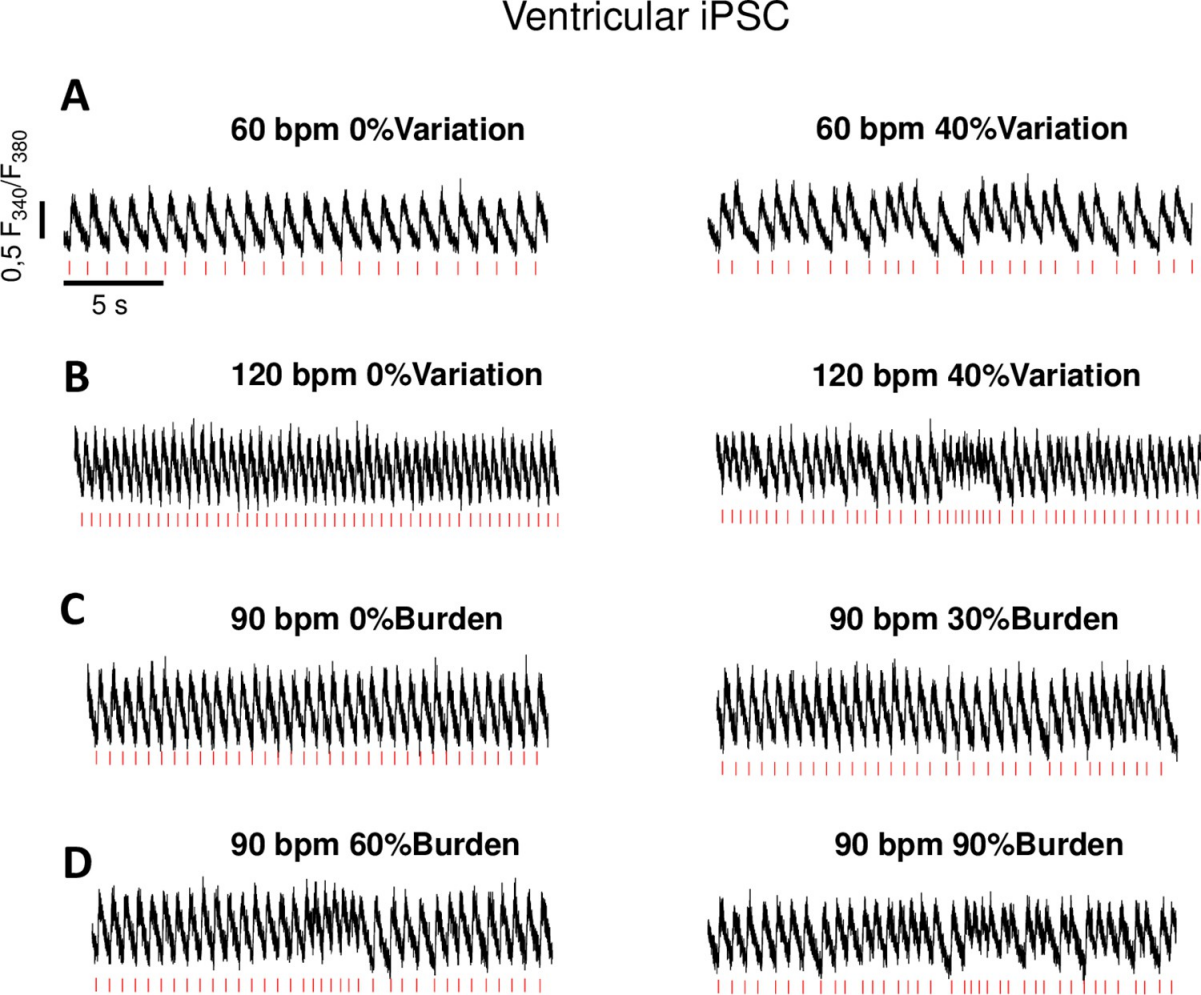
Original Ca^2+^ transient measurements of ventricular hiPSC-CM during simulation of cardiac arrhythmias. **(A)** Simulation of sinus rhythm (60 bpm) without arrhythmia and atrial fibrillation simulation at 60 bpm with beat-to-beat variation of 40%, **(B)** Tachycardia at 120 bpm without arrhythmia and tachycardia at 120 bpm with arrhythmia (40% beat to beat variation), **(C)** simulation at 90 bpm without arrhythmia burden and 30% arrhythmia burden, **(D)** simulation of 60% arrhythmia burden and 90% arrhythmia burden at 90 bpm.

**Fig 5:**
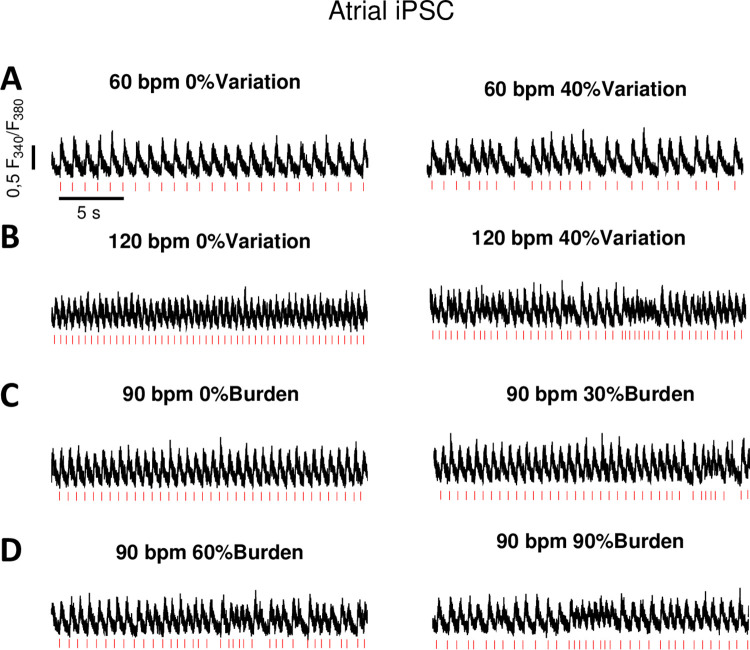
Original Ca^2+^ transient measurements of atrial hiPSC-CM during simulation of cardiac arrhythmias. **(A)** Simulation of sinus rhythm (60 bpm) without arrhythmia and atrial fibrillation simulation at 60 bpm with beat-to-beat variation of 40%, **(B)** Tachycardia at 120 bpm without arrhythmia and tachycardia at 120 bpm with arrhythmia (40% beat to beat variation), **(C)** simulation at 90 bpm without arrhythmia burden and 30% arrhythmia burden, **(D)** simulation of 60% arrhythmia burden and 90% arrhythmia burden at 90 bpm.

**Fig 6:**
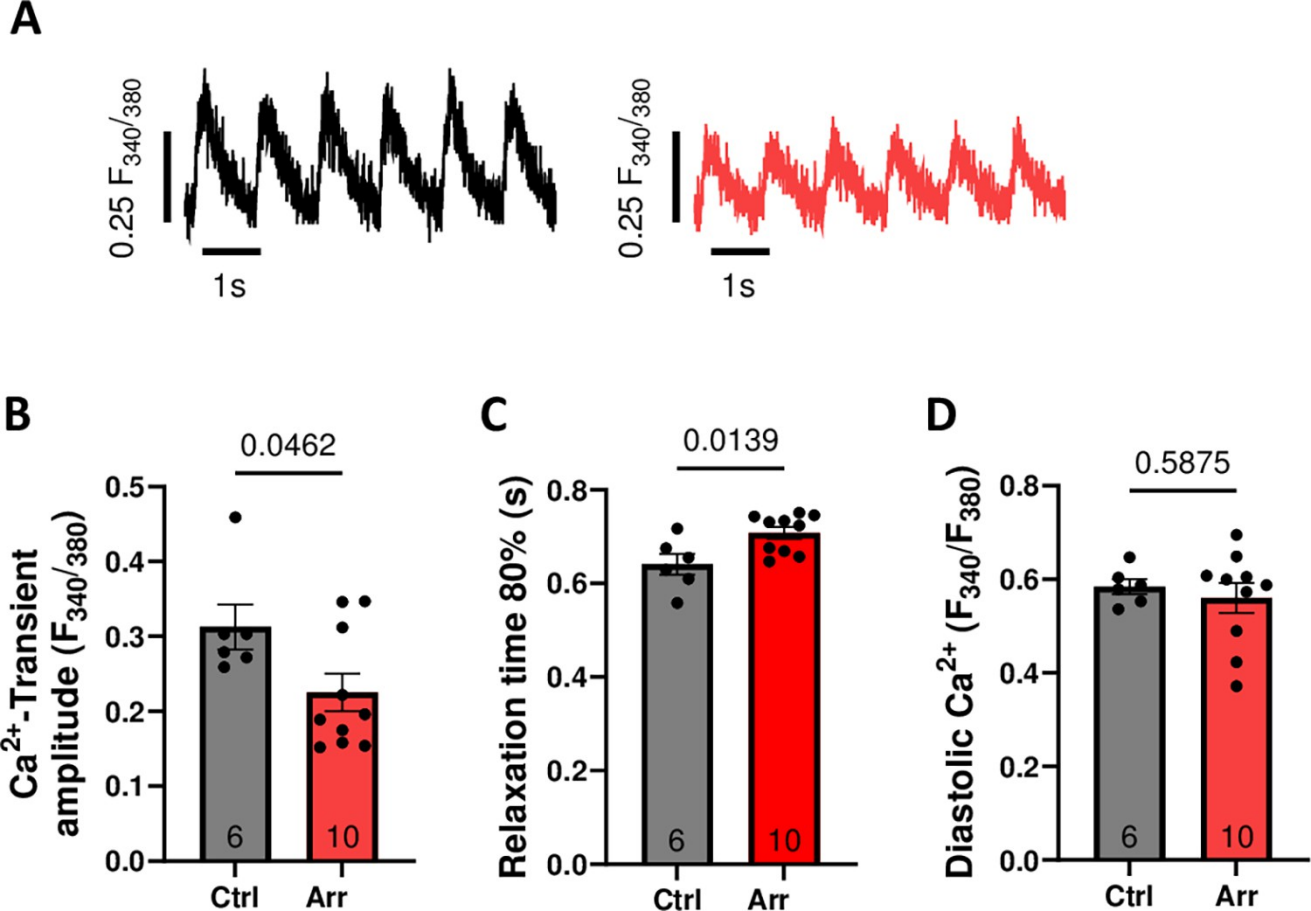
Atrial fibrillation (AF) simulation on atrial hiPS-CMs. Atrial hiPSC-CM underwent either AF simulation (arrhythmic pacing: Arr; 60 bpm, 40% beat-to-beat-variability) or rhythmic pacing (Ctrl; 60 bpm) chronically for 7 d. **(A)** Representative recordings of stimulated Ca^2+^ transients (epifluorescence microscopy, Fura-2) and **(B-D)** mean values for Ca^2+^ transient amplitude, relaxation time 80% and diastolic Ca^2+^ of atrial hiPSC-CM upon chronic AF simulation (Arr, n = 10 cardiomyocytes) or rhythmic pacing (Ctrl, n = 6). P-values were calculated using Student’s t-test.

AF is characterized by irregular RR intervals leading to arrhythmic ventricular excitation with or without tachycardia. Setting a beat-to-beat variation between the stimuli during culture pacing of hiPSC-CM (i.e. ventricular differentiated hiPSC-CM or other cell types) usually around 30–40% may mimic the typical clinical scenario. We recorded original traces of Ca^2+^ transients at the epifluorescence microscope and simulated normofrequent AF by stimulating ventricular and atrial hiPSC-CM at 60 bpm with a beat-to-beat variation of 40% (Figs 4A and 5A). The frequency might be adjusted accordingly. Importantly, in previous data, in vitro AF simulation in hiPSC-CM reflected the cellular electrophysiological phenotype of human ventricular myocardium from patients with AF [15]. We also tested the AF simulation in atrial hiPSC-CM (Fig 6A). After 7 days of AF simulation (60 bpm with 40% beat-to-beat variation) we observed reduced Ca^2+^ transient amplitude (Fig 6B: Control: 0.313±0.030 F_340/380_, n = 6 cells vs Arrhythmia: 0.225±0.025 F_340/380_, n = 10 cells) and prolonged Relaxation Time 80% (Fig 6C Control:0.641±0.022 s, Arrhythmia:0.708±0.013 s). Diastolic Ca^2+^ (Fig 6D) was not changed (Control: 0.585±0.016 F_340/380_, Arrhythmia:0.560±0.032 F_340/380_). These data are in line with changes in Ca^2+^ handling in atrial cardiomyocytes from patients with chronic AF [16].

### Tachycardia

The physiological mechanisms and the pathophysiological effects of chronic tachycardia are of clinical relevance. Simulating tachycardia chronically in hiPSC-CM offers a standardized and flexible model to study the mechanisms of tachycardia in different disease models including their time course. We simulated tachycardia by pacing at 120 bpm with and without arrhythmia and recorded the Ca^2+^ transients during stimulation (Figs 4B and 5B). We found that chronic tachycardic pacing up to 120 bpm is well feasible in hiPSC-CM over 7–10 days.

### Arrhythmia burden

Another possibility to use the culture stimulation model is to vary the time intervals of programmed stimulation. Thereby, different arrhythmia burden can mimic the clinical scenario of different AF burden, which is of prognostic relevance in patients [16]. Moreover, even the character of the arrhythmia can be varied (i.e. tachycardic episodes during AF). For instance, 1 h culture pacing can be divided into different parts with altered protocols. We simulated increasing arrhythmia burden starting with 0%, going on to 30%, 60% and 90% arrhythmia burden and recorded Ca^2+^ transients during the simulation to demonstrate the respective cardiomyocyte response (Figs 4C, 4D, 5C and 5D). In addition, premature ventricular contractions can also be simulated in that way in vitro. The diversity of other programmable designs is broad and allows the investigation of different clinical phenomena under controlled conditions in the laboratory. Importantly, the model facilitates the investigation of time-dependent progresses during arrhythmias.

### Translational relevance of in vitro simulation of arrhythmia in hiPSC-CM

The aim of the in vitro simulation of arrhythmias using hiPSC-CM is to mimic different rhythm disorders for a standardized translational investigation in a human model. This protocol might help to induce arrhythmia-associated phenotypes and to study mechanisms of arrhythmogenesis in atrial or ventricular hiPSC-CM, which are an established tool in many laboratories. HiPSC-CM after arrhythmia simulation can be used for various experimental readouts including transcriptomics, molecular biology or functional single cell studies. Importantly, hiPSC-CM undergoing the arrhythmia simulation protocol are suitable for genetic or pharmacological interventions including drug screening. Previously, we could demonstrate in different projects that in vitro arrhythmia simulation utilizing hiPSC-CM may resemble the clinical phenotype of AF patients [15,17]. As a comparison to hiPSC-CM after arrhythmia simulation, human myocardium from patients with normofrequent AF was investigated. Ventricular cardiomyocytes of these patients showed a reduction in the Ca^2+^ transient amplitude compared to ventricular cardiomyocytes from patients with sinus rhythm. Likewise, after ventricular hiPSC-CM underwent the in vitro arrhythmia simulation protocol for normofrequent AF (60 bpm with 40% beat-to-beat variation) cells were characterized by a significant reduced Ca^2+^ transient amplitude compared to rhythmically paced hiPSC-CM (60 bpm with 0% beat-to-beat variation) [15]. In atrial hiPSC-CM chronic AF simulation led to a decrease in Ca^2+^ transient amplitude and to prolonged relaxation time. These electrophysiological changes have also been observed in patients with chronic AF [16]. Arrhythmia simulation in hiPSC-CM could be further used for modeling a typical clinical scenario of different AF burden, which impacts mortality and hospitalization for heart failure after AF ablation [17]. HiPSC-CM with an AF burden >50% showed typical hallmarks of heart failure like decreased Ca^2+^ transient amplitudes, prolonged action potential duration and decreased sarcomere regularity [18–22]. Accordingly, a subanalysis of the CASTLE-AF trial showed that patients who had an AF Burden >50% had worsened heart failure outcomes [17]. Thus, the in vitro simulation of cardiac arrhythmias in hiPSC-CM may offer a versatile and translationally relevant tool for arrhythmia research.

### Step-by-step protocol, also available on protocols.io

S1 File provides the step-by-step protocol for the article ‘Simulation of cardiac arrhythmias in human induced pluripotent stem cell-derived cardiomyocytes’, also available on protocols.io (https://www.protocols.io/view/simulation-of-cardiac-arrhythmias-in-human-induced-kqdg32k1qv25/v1 ordx.doi.org/10.17504/protocols.io.kqdg32k1qv25/v1). The protocol on protocols.io is linked to PLOS ONE and is a peer-reviewed method.

## Supporting information

S1 Raw data (XLSX)

S1 File (PDF)
