# Supplementary material for: Simulation of cardiac arrhythmias in human induced pluripotent stem cell-derived cardiomyocytes
Source: PLoS One. 2024 Sep 27;19(9):e0310463. doi: 10.1371/journal.pone.0310463 (PMC11432883; doi:10.1371/journal.pone.0310463)
Supplement: S1 File — (PDF) [file pone.0310463.s002.pdf]

Sep 06, 2024

Simulation of cardiac arrhythmias in human induced pluripotent stem cell-derived cardiomyocytes

Peer-reviewed method

DOI
dx.doi.org/10.17504/protocols.io.kqdg32k1qv25/v1

Thea Bommer[1], Maria Knierim[2], Julia Unsöld[3], Dominic Riedl[4], Laura Stengel[1], Michael Paulus[1], Thomas Körtl[4], Norman Liaw[5], Lars S. Maier[1], Katrin Streckfuss-Bömeke[3,6], Samuel Sossalla[4], Steffen Pabel[1,7]

[1]Department of Internal Medicine II, University Hospital Regensburg, Regensburg, Germany;
[2]Department of Cardiothoracic and Vascular Surgery, University Medical Centre Göttingen, Göttingen, Germany;
[3]Institute of Pharmacology and Toxicology, University of Würzburg, Würzburg, Germany;
[4]Justus-Liebig-University Gießen Medical Clinic I and Campus Kerckhoff Bad Nauheim, Gießen and Bad Nauheim, Germany;
[5]Institute of Pharmacology and Toxicology, University Medical Centre Göttingen, Göttingen, Germany;
[6]Clinic for Cardiology and Pneumology, Georg-August University Göttingen, and DZHK (German Centre for Cardiovascular Research), partner site Göttingen, Germany;
[7]Center for Systems Biology, Massachusetts General Hospital and Harvard Medical School, Boston, MA, USA

PLOS ONE Lab Protocols
Tech. support email: **plosone@plos.org**

Thea Bommer
University Clinic Regensburg Internal Med II

OPEN ACCESS

DOI: dx.doi.org/10.17504/protocols.io.kqdg32k1qv25/v1

Protocol Citation: Thea Bommer, Maria Knierim, Julia Unsöld, Dominic Riedl, Laura Stengel, Michael Paulus, Thomas Körtl, Norman Liaw, Lars S. Maier, Katrin Streckfuss-Bömeke, Samuel Sossalla, Steffen Pabel 2024. Simulation of cardiac arrhythmias in human induced pluripotent stem cell-derived cardiomyocytes. **protocols.io** https://dx.doi.org/10.17504/protocols.io.kqdg32k1qv25/v1

License: This is an open access protocol distributed under the terms of the **Creative Commons Attribution License**, which permits unrestricted use, distribution, and reproduction in any medium, provided the original author and source are credited

Protocol status: Working
We use this protocol and it's working

Created: June 20, 2024

Last Modified: September 06, 2024

Protocol Integer ID: 102344

Keywords: iPSC cardiomyocytes, arrhythmias, electrophysiology, culture pacing, translational models

Abstract

The effects and mechanisms of cardiac arrhythmias are still incompletely understood and an important subject of cardiovascular research. A major difficulty for investigating arrhythmias is the lack of appropriate human models. Here, we present a protocol for a translational simulation of different types of arrhythmias using human induced pluripotent stem cell-derived cardiomyocytes (hiPSC-CM) and electric cell culture pacing. The protocol comprises the handling of ventricular and atrial hiPSC-CM before and during in vitro arrhythmia simulation and possible arrhythmia simulation protocols mimicking clinical arrhythmias like atrial fibrillation. Isolated or confluent hiPSC-CM can be used for the simulation. In vitro arrhythmia simulation did not impair cell viability of hiPSC-CM and could reproduce arrhythmia associated phenotypes of patients. The use of hiPSC-CM enables patient-specific studies of arrhythmias, genetic interventions, or drug-screening. Thus, the in vitro arrhythmia simulation protocol may offer a versatile tool for translational studies on the mechanisms and treatment options of cardiac arrhythmias.

Guidelines

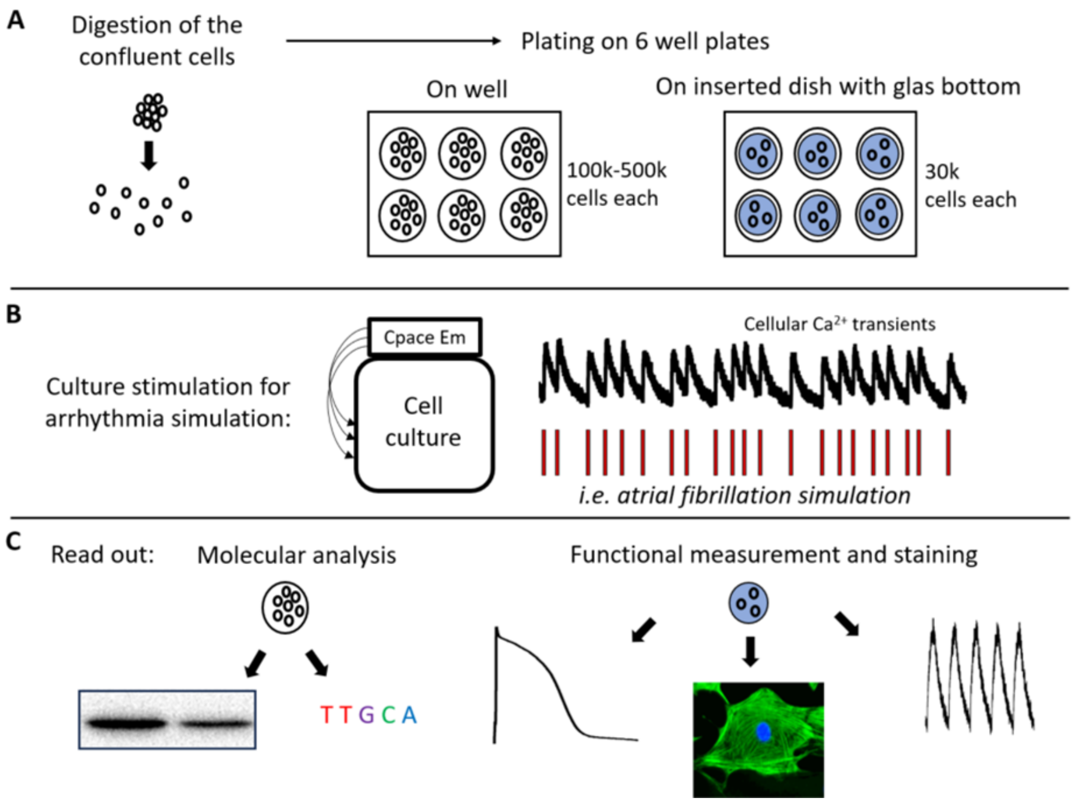

Summary of the protocol for in vitro arrhythmia simulation using hiPSC-CM.
(A) Confluent grown hiPSC-CM are digested and plated depending on the planned experimental read-out on 6 well plates (100 000-500 000 cells per well i.e. for acquisition of cell pellets) or on glass bottom dishes (30 000 cells per dish inserted in the 6 well plates i.e. for measuring single cells). **(B)** After plating of the cells, the electrodes of the C-Pace EM stimulation system are placed as lid on the 6-well dishes and arrhythmia simulation is conducted. **(C)** After arrhythmia simulation, cells can be directly used for respective experiments.

Materials

Equipment and materials:

- The fixed devices that are used for preparation and the culture stimulation of hiPSC-CM:

A	B	C
**Device**	**Producer**	**Identification**
Incubator (37°C, 5% CO2)	Heraeus	Heracell 240i
Culture Pacer	IonOptix	C-Pace EM Channel
Electrodes	IonOptix	C-Dish
Ribbon Cable 300 V 10 fiber	IonOptix	AWM2651105C

- All consumables:

A	B	C
**Consumable**	**Producer**	**Item number**
T25 cell culture bottle	Sarstedt	83.3910.002
6 well Falcon tissue culture plate	Corning	353046
Glass bottom dish, 35 mm (i.e. Fluoro Dish™)	WPI	FD35-100
Centrifuge tube	Sarstedt	62.554.502
Reaction tube	Eppendorf	variable
Serological pipettes	Sarstedt	86.1253.025
2 mL Pasteur pipettes	Roth	4522.1
50 mL Steriflip-GP	Merck	SCGP00525

Cell culture flask, T-25 **Sarstedt Catalog #**83.3910.500

Falcon® 6-well Clear Flat Bottom TC-treated Multiwell Cell Culture Plate with Lid Individually Wra **Corning Catalog #**353046

Fluorodish **World Precision Instruments Catalog #**FD35-100

Tube 15ml 120x17mm PP **Sarstedt Catalog #**62.554.502

Serological pipette **Sarstedt Catalog #**86.1253.025

Pasteur pipettes, 2 ml **CarlRoth Catalog #**4522.1

Steriflip-GP Sterile Centrifuge Tube Top Filter Unit **Merck Millipore (EMD Millipore) Catalog #**SCGP00525

- The reagents for the preparation of the plates and the solutions for differentiation, culturing and digesting the cells:

A	B	C
**Reagent**	**Producer**	**Item number**
Gelatine	Sigma Aldrich	G1890
Aqua irrigation solution	B Braun	0082479E
RPMI 1640 (1x) +GlutaMAX + HEPES	Gibco	72400-021

A	B	C
L-Ascorbic Acid 2-Phosphate	Sigma Aldrich	A8960
Albumin	Sigma Aldrich	A9731
CHIR99021	Millipore	361559
Inhibitor of Wnt production-2	Millipore	681671
Retinoic acid	Sigma Aldrich	R2625
B27 Supplement	Gibco	17504-044
Lactate	Sigma Aldrich	L4263
FBS Qualified	Gibco	A3160802
Trypsin-EDTA (0.25%)	Gibco	25200-056
Thiazovivin, iPSC Induction Enhancer	Merck	420220-10MG
DMSO	Sigma	D2650-100ML

Gelatin from porcine skin **Merck MilliporeSigma (Sigma-Aldrich) Catalog #**G1890

RPMI 1640 Medium, GlutaMAX™ Supplement, HEPES **Gibco - Thermo Fisher Catalog #**72400021

B-27 Supplement **Gibco - Thermo Fischer Catalog #**17504044

Fetal Bovine Serum, qualified, One Shot™ format, Brazil **Thermo Fisher Catalog #**A3160802

Trypsin-EDTA (0.25%), phenol red **Thermofisher Catalog #**25200-056

iPSC Induction Enhancer, Thiazovivin **Merck MilliporeSigma (Sigma-Aldrich) Catalog #**420220-M

Dimethyl sulfoxide 100mL **Merck MilliporeSigma (Sigma-Aldrich) Catalog #**D2650-100ML

- The medium used for differentiation of the hIPSC-CM:

A	B	C	D
**Substance**	**Concentration**	**Producer**	**Item number**
L-Ascorbic Acid 2-Phosphate	200 mg/L	Sigma Aldrich	A8960
Albumin, human recombinant	500 mg/L	Sigma Aldrich	A9731
CHIR99021	4 µMol/L	Millipore	361559
Solved in RPMI 1640 (1x) + GlutaMAX + HEPES		Gibco	72400-021

- The medium used for culturing the hiPSC-CM:

A	B	C	D
**Substance**	**Concentration**	**Producer**	**Item number**
B27 Supplement	2%	Gibco	17504-044
Solved in RPMI 1640 (1x) +GlutaMAX + HEPES		Gibco	72400-021

B-27 Supplement **Gibco - Thermo Fischer Catalog #**17504044

RPMI 1640 Medium, GlutaMAX™ Supplement, HEPES **Gibco - Thermo Fisher Catalog #**72400021

- The composition of the digestion medium for the hiPSC-CM digestion before plating:

A	B	C	D
**Substance**	**Concentration**	**Producer**	**Item number**
FBS Qualified	20%	Gibco	A3160802
Thiazovivin, iPSC Induction Enhancer	0.13%	Merck	420220-10MG
Solved in Culture medium hiPSC-CM			

Fetal Bovine Serum, qualified, One Shot™ format, Brazil **Thermo Fisher** **Catalog #**A3160802

iPSC Induction Enhancer, Thiazovivin **Merck MilliporeSigma (Sigma-Aldrich)** **Catalog #**420220-M

- Thiazovivin solution that is added to the hiPSC-CM culture medium:

A	B	C	D
**Substance**	**Concentration/Volume**	**Producer**	**Item number**
Thiazovivin	10 mg	Merck	420220-10MG
DMSO	16,06 mL	Sigma	D2650-100ML

iPSC Induction Enhancer, Thiazovivin **Merck MilliporeSigma (Sigma-Aldrich)** **Catalog #**420220-M

Dimethyl sulfoxide 100mL **Merck MilliporeSigma (Sigma-Aldrich)** **Catalog #**D2650-100ML

Differentiation of ventricular and atrial hiPSC-CM

1 Differentiation of hiPSC-CM was performed as previously described [1,2]. Briefly, hiPSCs were cultured feeder-free and adherent on cell culture dishes in the presence of chemically defined medium E8.

2 Cardiac differentiation of hiPSCs was performed by sequential targeting of the WNT pathway. Undifferentiated hiPSCs were cultured as a monolayer on Geltrex-coated 12-well dishes to a confluence of 85%-95%.

3 Medium was changed to cardio differentiation medium composed of RPMI medium supplemented with L-Ascorbic Acid 2-Phosphate and albumin including the GSK3 inhibitor CHIR99021 (d0).

4 After 48h, medium was changed to fresh media supplemented with 5 µmol/L of the inhibitor of Wnt production-2 for two days. For atrial subtype specification, 1 µmol/L retinoic acid was added between day 3 and day 6 [3].

5 Cardiac contraction is usually observed on day 7. From day 8 (atrial) or day 10 (ventricular), the cells were cultured in cardio culture medium, which was changed every 2-3 days.

6 HiPSC-CM were purified after 20-30 (atrial) or 20-40 (ventricular) days of differentiation by metabolic selection for 4-5 days using lactate (4 mmol/L, Sigma Aldrich) as carbon source. In contrast to undifferentiated iPSCs the differentiated cardiomyocytes have the ability to effectively uptake and use lactate as a carbon source [4]. Thereby, substitution of glucose by lactate allows differentiated cardiomyocytes to grow whereas undifferentiated iPSCs die.

7 Following differentiation, purity of hiPSC-CM was determined by flow analysis (~90% cardiac troponin T +), cardiac immuno-fluorescence, morphology and qPCR for cardiac sub-type marker as previously described [1, 2, 5, 6].

Preparation of the 6 well culture plates and dishes

1h 20m

8

> **Note**
>
> Depending on the experimental purpose of the cells (single cell readouts or usage of cell pellets), hiPSC-CM are digested and plated either into 6 wells or glass bottom dishes as described below. Before plating, the dishes or wells need to be coated with gelatin (or other respective agents) ensuring optimal adherence of the cells:

Prepare 0.1 % gelatin solution in distilled water.

9 Autoclave at 120 °C and 1 bar for 00:20:00 . 20m

10 Coat plates with 1 mL gelatin solution per well or dish.

11 Incubate coated plates either Overnight at 4 °C or for 00:30:00 at Room temperature 30m

12 Extract the fluid supernatant.

13 Dry plates for a minimum 00:30:00 until no wetness is left in the wells or dishes. 30m

Note

Coated plates are savable up to 7 days in the incubator.

Digestion and plating of the hiPSC-CM 15m

14

Note

HiPSC-CM at the age of 60-70 days are used for in vitro arrhythmia simulation.

For plating the cells, we perform a digestion protocol using trypsin as described before [2]. Briefly:

15 Add 1 mL - 2 mL Trypsin to confluent hiPSC-CM cultures.

16 Incubate for 00:03:00 - 00:10:00 at 37 °C . 10m

17 Stop reaction by adding 2 mL of the digestion medium when cells dissolve.

18 Transfer suspension into a tube that already contains 2 mL digestion medium.

19 Centrifuge at 200 x g, Room temperature, 00:05:00 . 5m

20 Extract supernatant and resuspend the pellet in digestion medium.

21 Count cells (i.e. via Neubauer chambers) and distribute them according to the targeted cell numbers in coated plates.

22 Plating is performed based on the planned experiments:

22.1 For assays requiring larger cell numbers (100 000 – 500 000 cells, i.e. Western Blots, Proteomics or gene sequencing) plate hiPSC-CM in the wells of the 6 well plate.

22.2 For investigation of single cells (i.e. patch clamp, fluorescence microscopy), plate a cell number of 20 000 – 40 000 hiPSC-CM in 35 mm glass bottom dishes, which are placed within the wells of the 6 well plate.

23 Fill every well with 2 mL and every dish with 1.5 mL digestion medium.

24 Resuspend cells very gentle to avoid damaging the cells and add needed cell number to the prepared medium.

Note

For better distribution of the cells move the plate horizontally and vertically (i.e. '8-shape')

25 After 2 days: replace the digestion medium by culture medium

Note

After plating conduct a 7-day resting period for recovery and adhering of the cells before starting the stimulation protocol.

26 Change medium every two days.

Note

By plating the hiPSC-CM in 35 mm glass bottom dishes, which are placed within the wells of the 6 well plate, cells could directly be transferred after stimulation within the dish to respective experimental setups.

Electrode preparation

20m

27 Electrode preparation and cleaning of the electrodes is described in the manufacturer's guidelines to ensure full technical support. We prepared the electrodes as follows:

27.1 Autoclave electrodes at 120 °C for 00:20:00 at 1 bar. 20m

27.2 After cleaning, the electrodes can be placed on 6 wells with or without inserted glass bottom dishes.

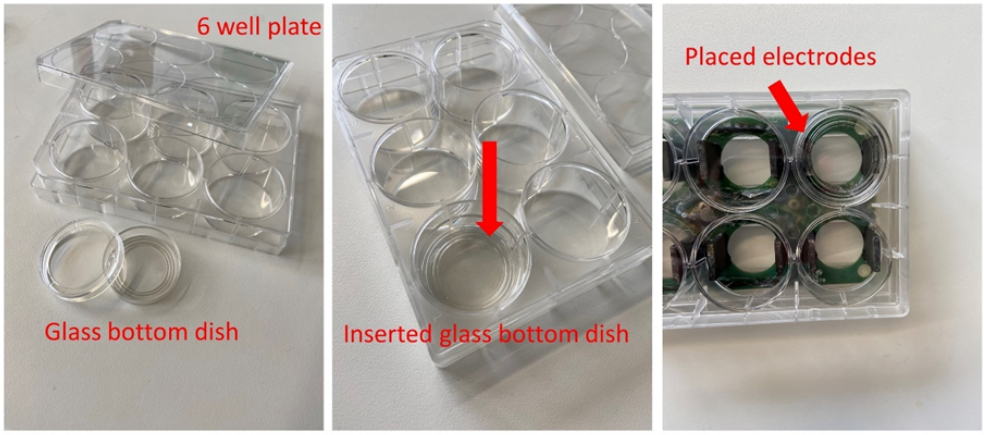

Placing of glass bottom dishes and stimulation electrodes. Single glass bottom dishes (left panel) that fit right into single wells of the 6 well plate can be inserted in the 6 well plate (middle panel). The electrodes fit for both 6 well plate and inserted glass bottom dishes (right panel). Therefore, cells can be directly transferred to functional studies after in vitro arrhythmia simulation.

28

Note

To autoclave the electrodes, we cover the components of the circuit board on the back of the electrodes with silicon. Thereby, electrodes can be protected during autoclaving.

The single glass bottom dishes fit into the 6 well but still leave enough space for the electrodes. Therefore, direct measurements of single cells without another digestion/plating can be conducted after arrhythmia simulation since the dishes can be directly transferred to the microscope.

Setting the electrical stimulus pulse

7h 30m

29 After plating and resting, electrical stimulation of the cells was started:

30 Place clean electrodes into the wells in the sterile bench.

31 Connect each plate via ribbon cable to one channel of the pacer.

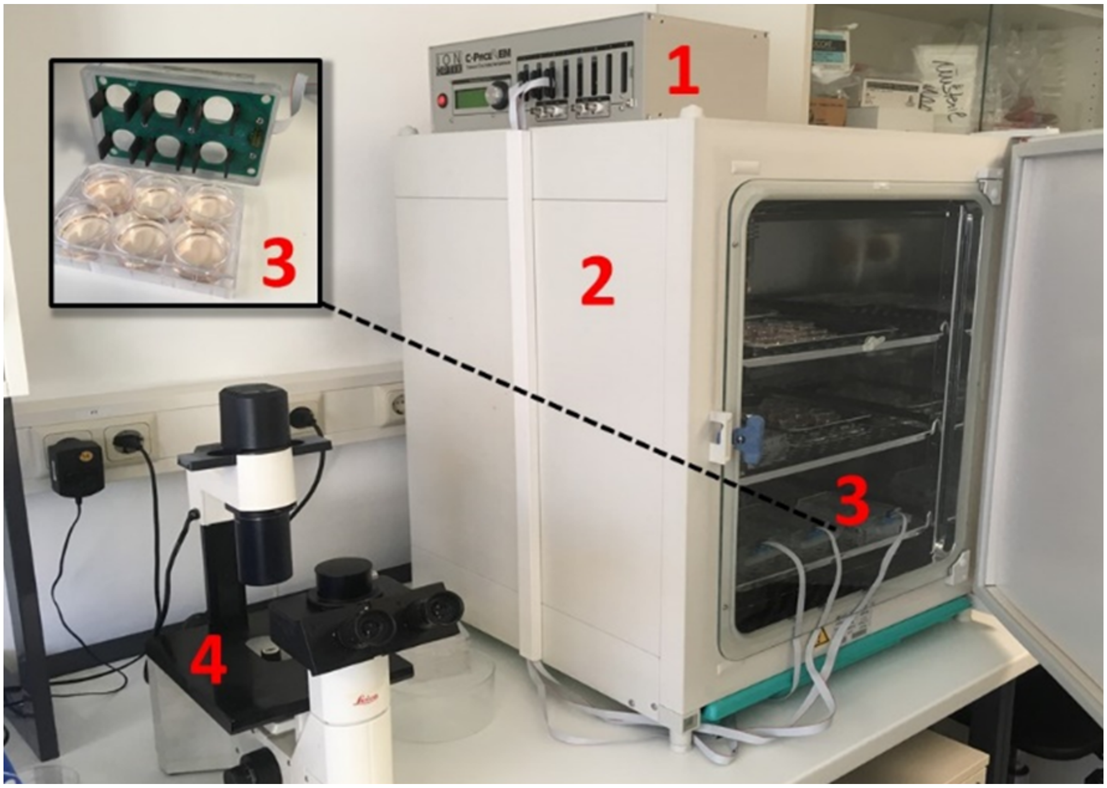

Exemplary setup for the culture stimulation of hiPSC-CM. The C-Pace EM System (IonOptix) (1) on top of the incubator (2) with output via ribbon cables connecting the pacer to the electrodes in the 6 well plates with hiPSC-CM (3). Sufficient capture and contraction of hiPSC-CM are monitored via microscope (4).

32 Stimulate every plate individually according to the planned experiments by programming the single channels of the pacer.

33 Adjust the stimulation pulse for starting the pacing.

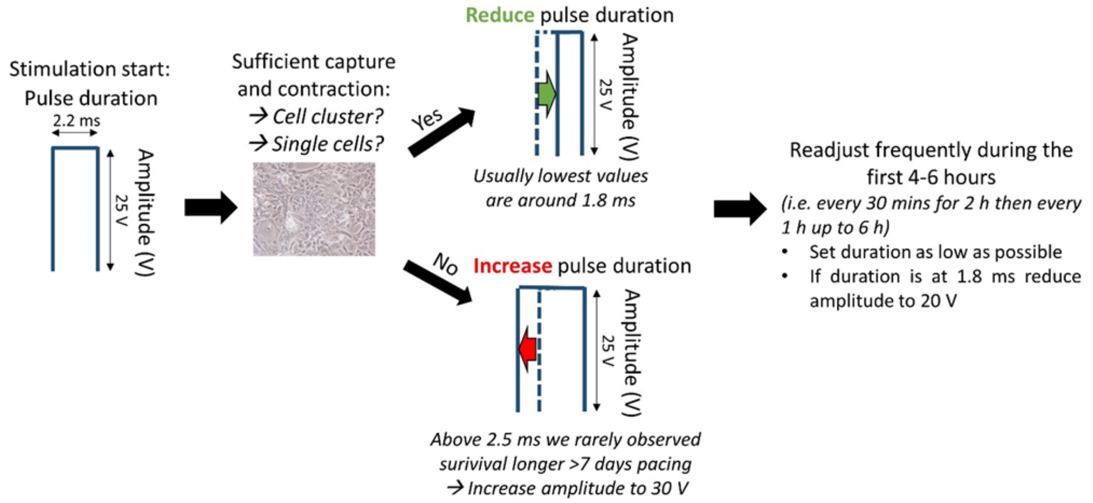

Setting the stimulus pulse. When starting the culture stimulation, we recommend using stimulus pulses with a duration of 2.2 ms with an amplitude of 25 V. The capturing and contraction of the cell clusters and the single cells need to be evaluated under the microscope. If capturing and contraction are sufficient, pulse duration should be reduced stepwise. If capturing and contraction are not sufficient, pulse duration needs to be increased to ensure appropriate pacing. Constant readjustment of the pulse parameters, especially during the first 4-6 hours is required. Set stimulation pulse around 10% above threshold.

34 Control the contraction of the cells under the microscope:

34.1 Right at the start of the stimulation.

34.2 For the first two hours: frequently repeated, at least every 00:30:00 . 30m

34.3 At the first day: control/readjust the stimulation for around every 01:00:00 for the following 06:00:00 . 7h

34.4 The following days: check cells for vitality (typical shape? detachment?) and visible contraction of the cardiomyocytes minimum two times a day.

34.5 Protocol pacing settings and cell contraction (i.e. percentage of beating cardiomyocyte in a prespecified area).

Note

The stimulus pulses are square pulses consisting of pulse amplitude (V) and duration (ms). Pulses need to be sufficient to excite the contraction of the cells, but as low as possible to not cause damage to the cells and thereby impair their physiology. When setting the pulse parameters, pulse duration is set as short as possible. To ensure the right pulse strength and duration the contraction of the cells is frequently controlled under the microscope during the stimulation, especially at the beginning of the stimulation protocol. Cells usually reach a steady state after 1 day of pacing.

Medium changes

35

Note

Frequency of the medium changes depends on the density of the cells: in dishes with a cell number of around 30 000 stimulated cells change medium two times a week, in wells with 100 000 plated cells or more change medium three times a week

Disinfect all material that is used thoroughly with ethanol.

36 Warm up fresh culture medium at 37 °C .

37 Remove the electrodes together with the top of the 6 well plate under the sterile working bench.

38 Remove old medium.

39 Add 2 mL fresh medium per well and 1.5 mL per dish.

Note

Medium is carefully pipetted in the inserted dishes. Fluids between the walls of the 6 well plate and the inserted glass bottom dish could hinder the removal of the dish from the 6-well plate.

Monitoring of apoptosis during culture pacing

40 To evaluate if the cells get stressed or damaged during culture pacing we perform assays for detecting apoptosis. One assay we used is the APC Annexin V Apoptosis Detection Kit (BioLegend). It was used as previously described [5,7]. In short:

41 Dissociate cells from the culture plates.

42 Expose cells to Annexin V Binding Buffer.

43 Analyse apoptosis by flow cytometry with dual excitation at 488 nm and 635 nm.

44 Gate events using a positive control with staurosporine (1 mmol/l).

45 Calculate apoptosis rate as the percentage of events positive for APC and negative for propidium iodide.

Note

Moreover, methylene blue stainings were performed.

Protocol references

References:

1. Borchert T, Hübscher D, Guessoum CI, Lam TD, Ghadri JR, Schellinger IN, et al. Catecholamine-Dependent β-Adrenergic Signaling in a Pluripotent Stem Cell Model of Takotsubo Cardiomyopathy. J Am Coll Cardiol. 2017;70(8):975-91.

2. Haupt LP, Rebs S, Maurer W, Hübscher D, Tiburcy M, Pabel S, et al. Doxorubicin induces cardiotoxicity in a pluripotent stem cell model of aggressive B cell lymphoma cancer patients. Basic Res Cardiol. 2022;117(1):13.

3. Kleinsorge M, Cyganek L. Subtype-Directed Differentiation of Human iPSCs into Atrial and Ventricular Cardiomyocytes. STAR Protoc. 2020;1(1):100026.

4. Tohyama S, Hattori F, Sano M, Hishiki T, Nagahata Y, Matsuura T, et al. Distinct metabolic flow enables large-scale purification of mouse and human pluripotent stem cell-derived cardiomyocytes. Cell Stem Cell. 2013;12(1):127-37.

5. Pabel S, Knierim M, Stehle T, Alebrand F, Paulus M, Sieme M, et al. Effects of Atrial Fibrillation on the Human Ventricle. Circ Res. 2022;130(7):994-1010.

6. Waas M, Weerasekera R, Kropp EM, Romero-Tejeda M, Poon EN, Boheler KR, et al. Are These Cardiomyocytes? Protocol Development Reveals Impact of Sample Preparation on the Accuracy of Identifying Cardiomyocytes by Flow Cytometry. Stem Cell Reports. 2019;12(2):395-410.

7. Paulus MG, Renner K, Nickel AG, Brochhausen C, Limm K, Zügner E, et al. Tachycardiomyopathy entails a dysfunctional pattern of interrelated mitochondrial functions. Basic Res Cardiol. 2022;117(1):45.
